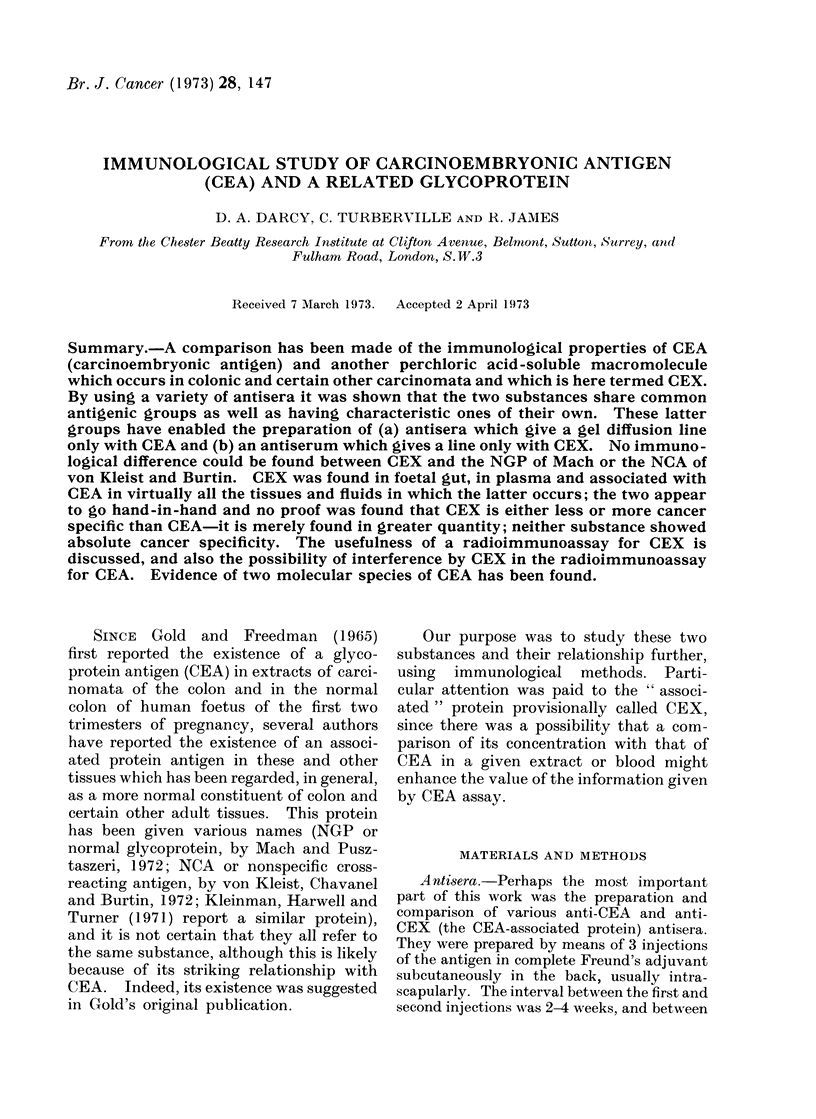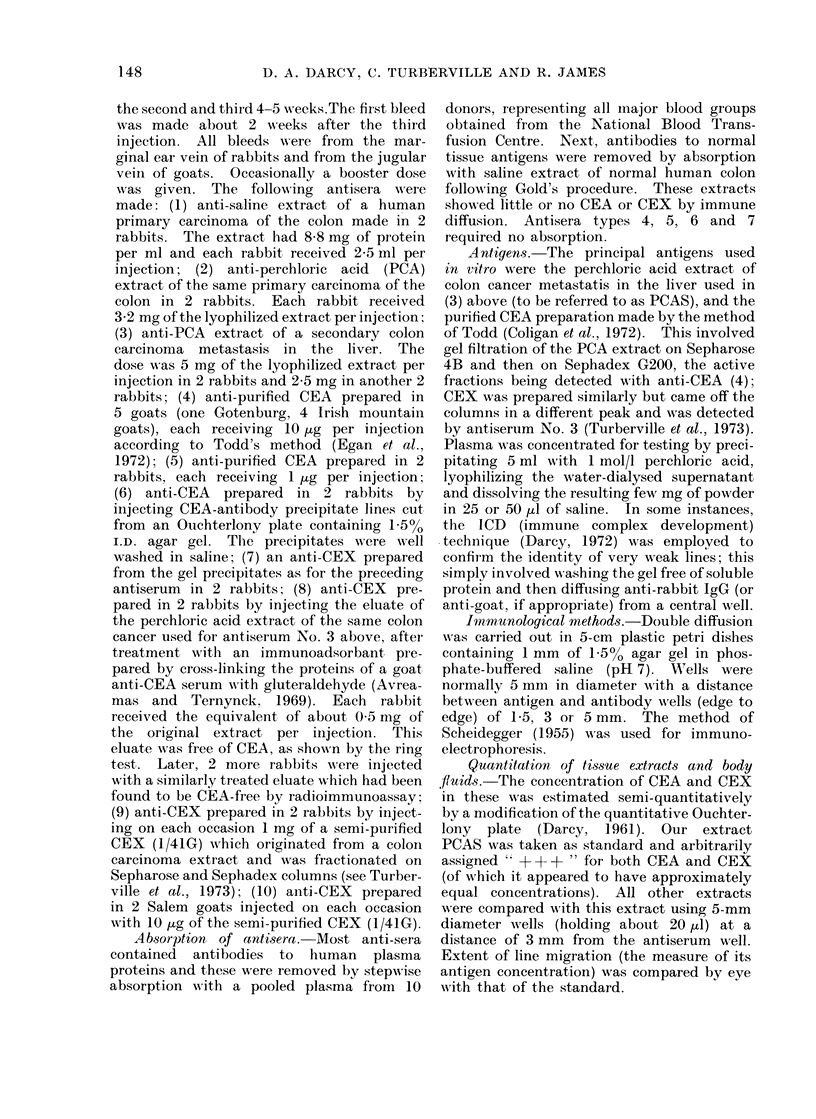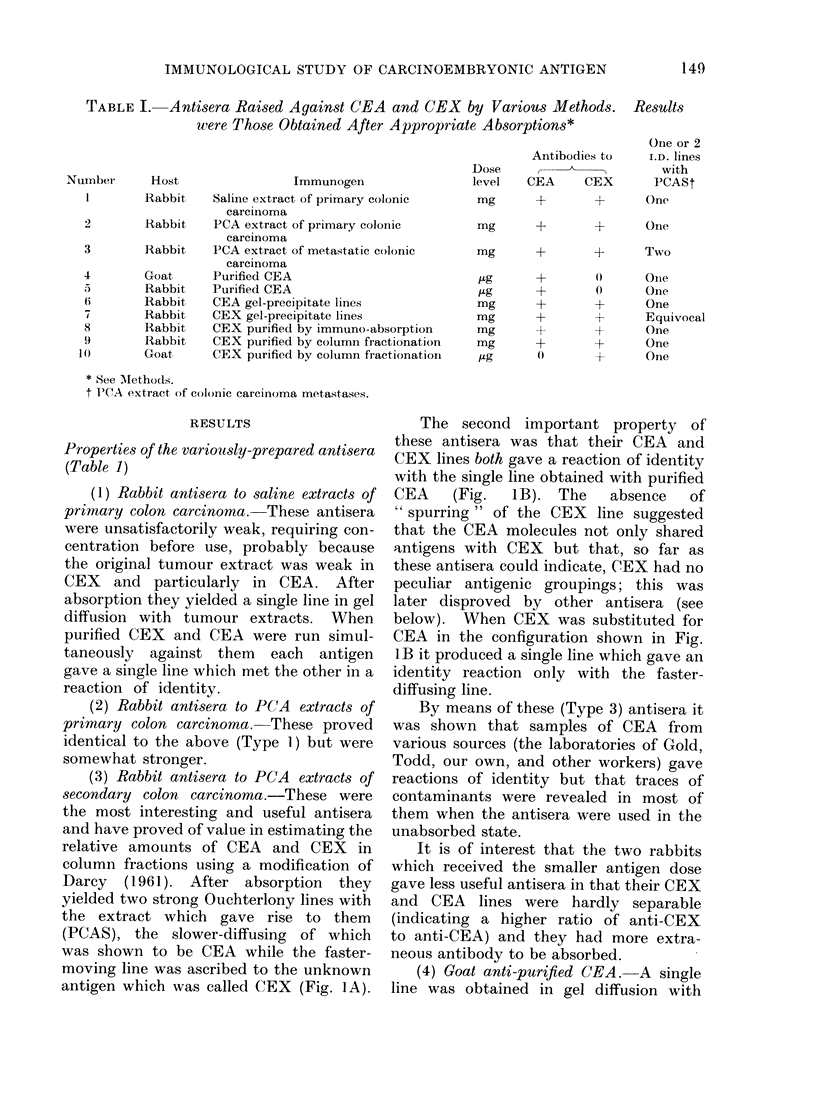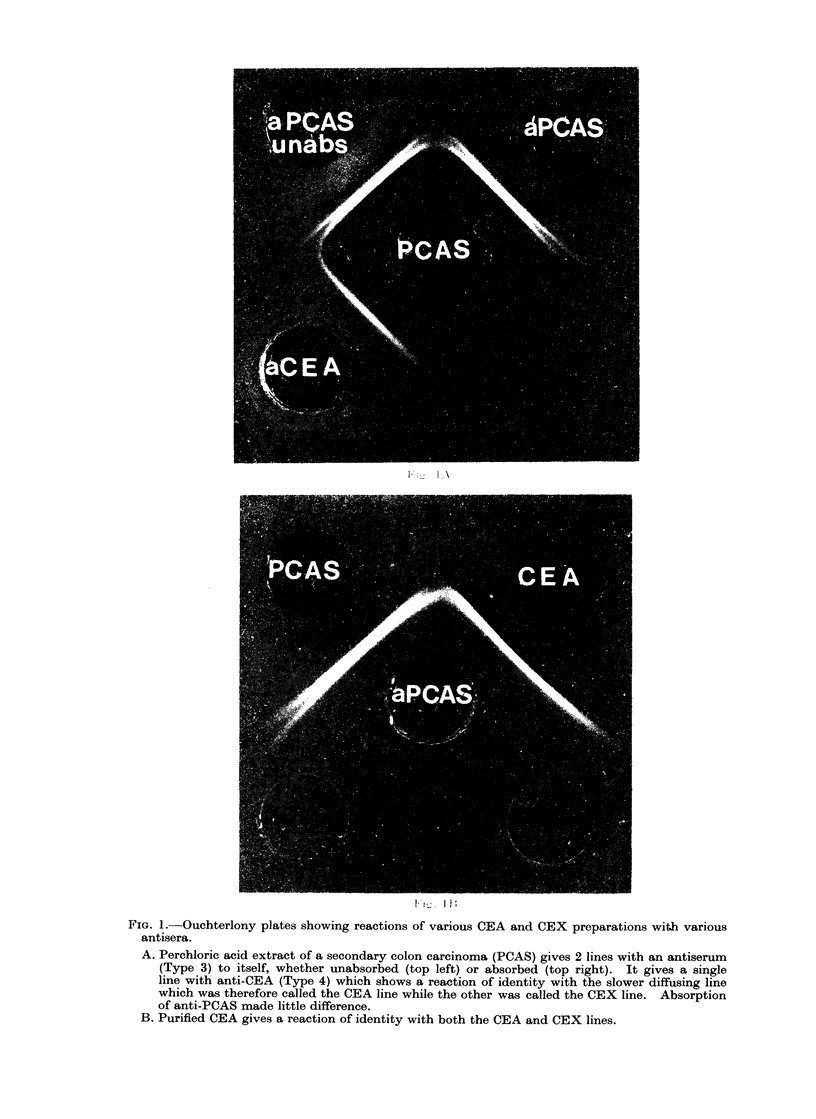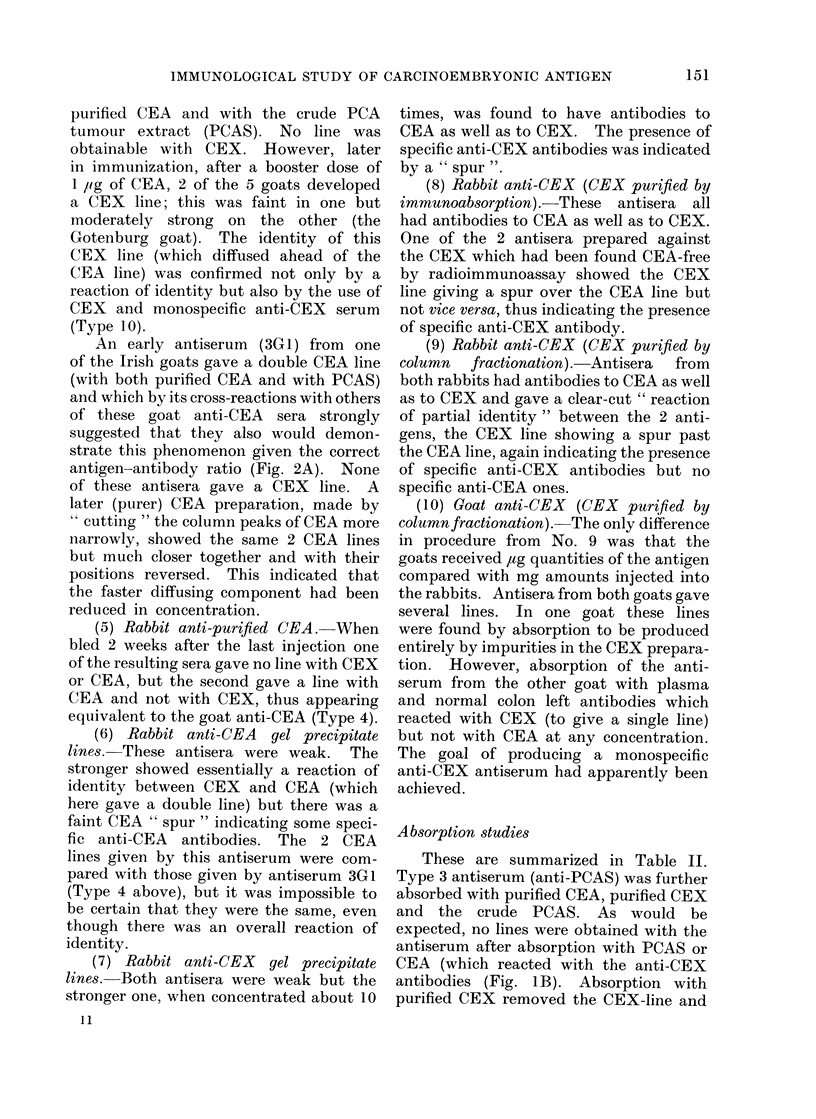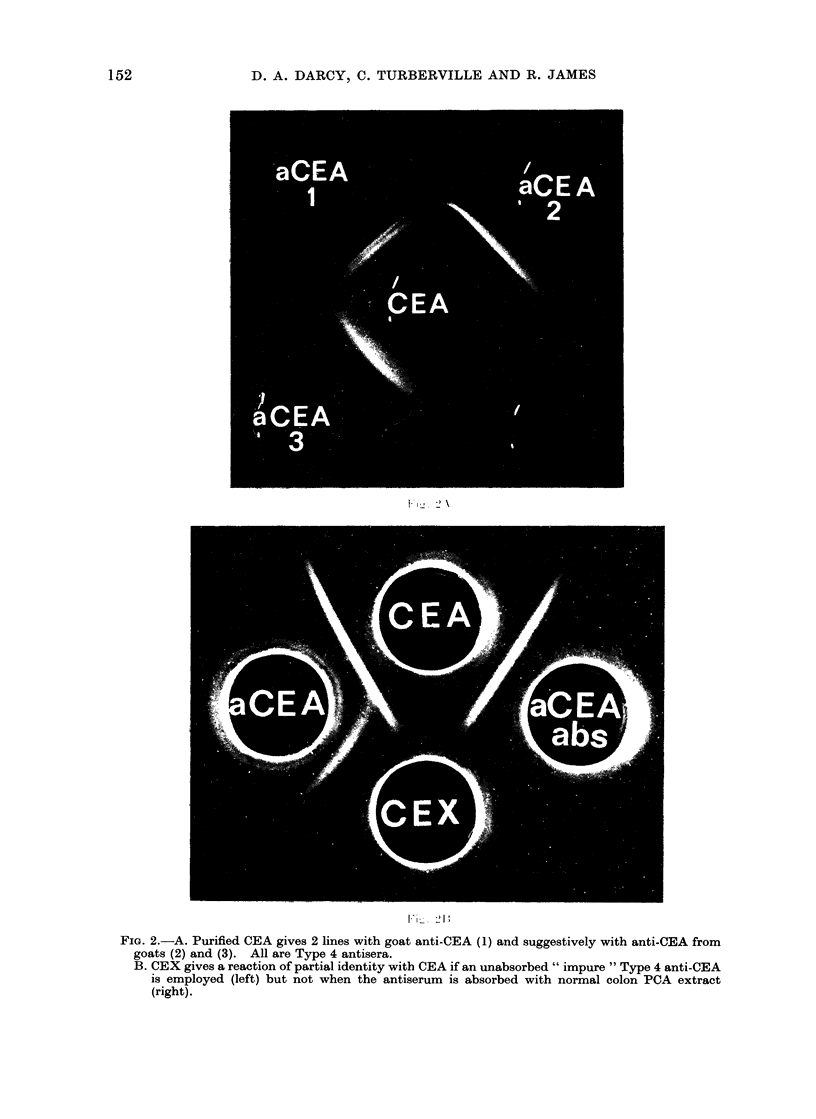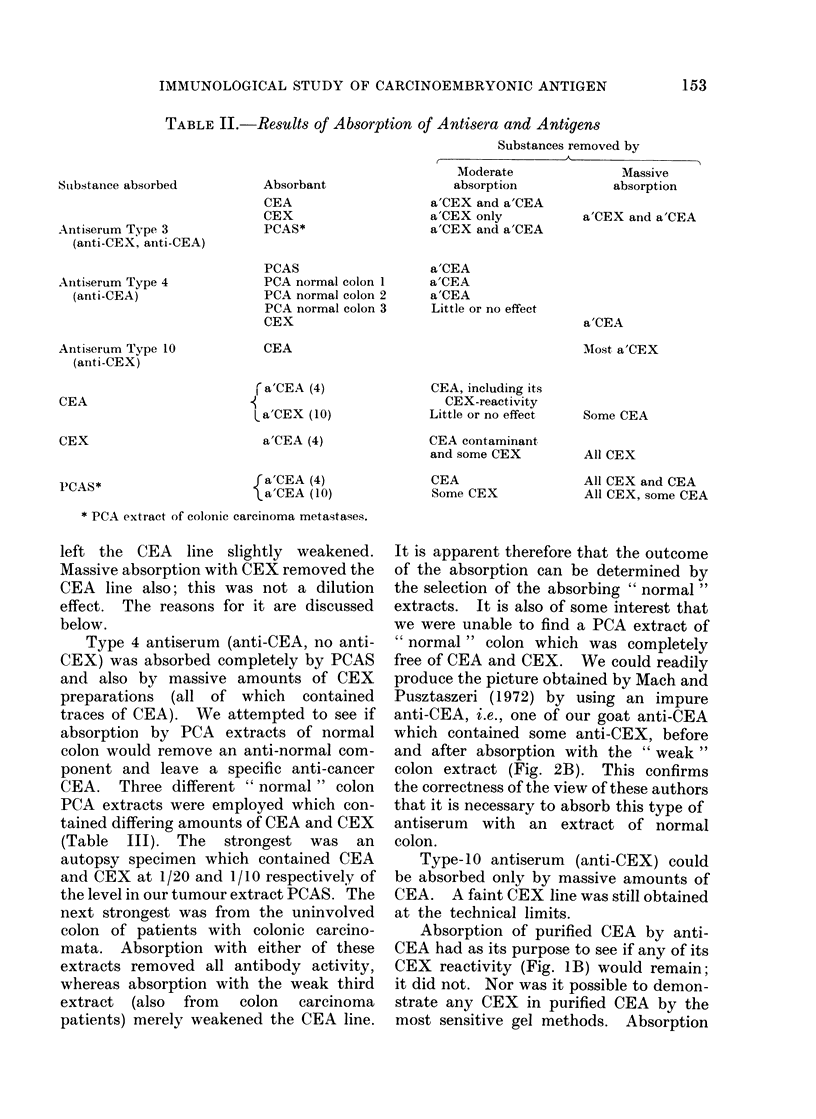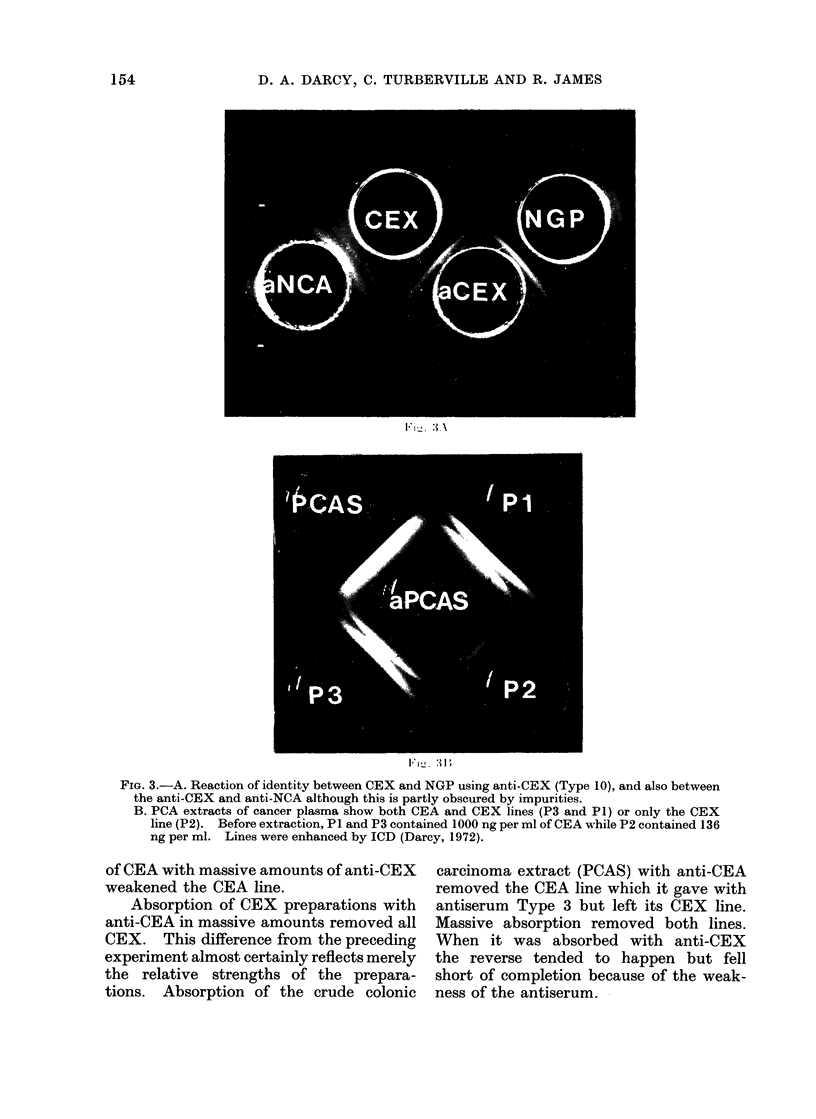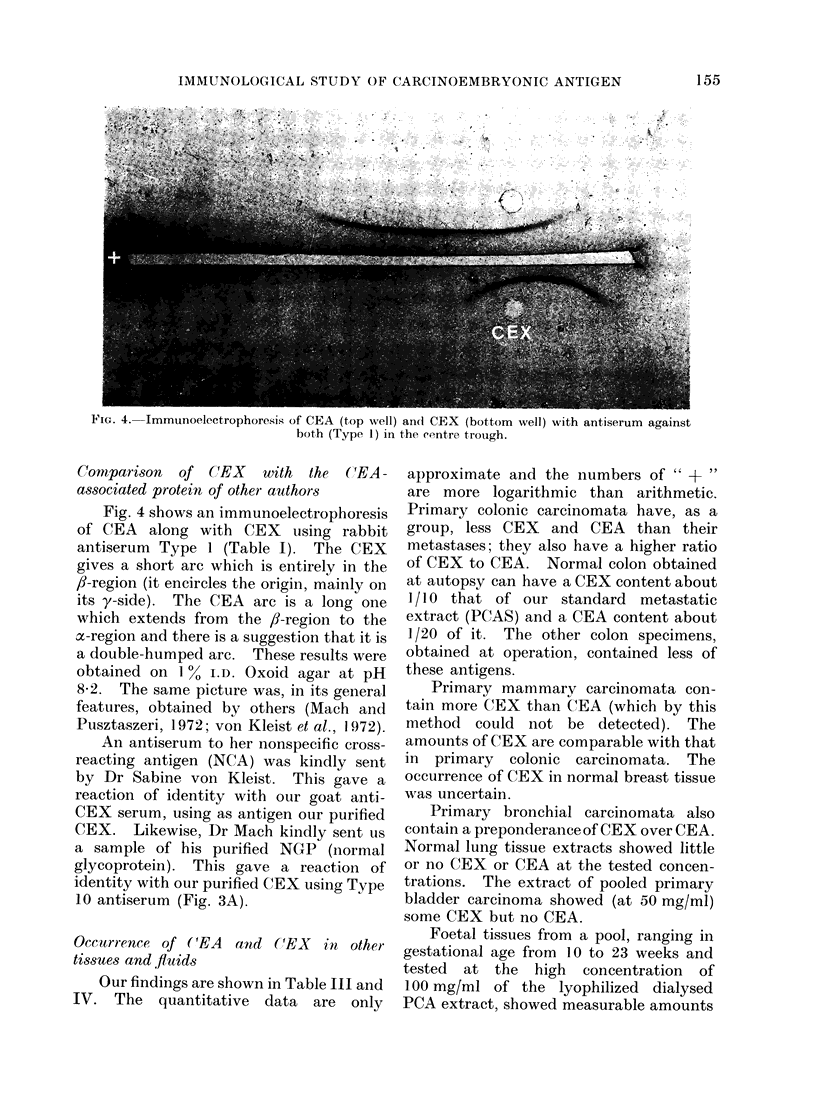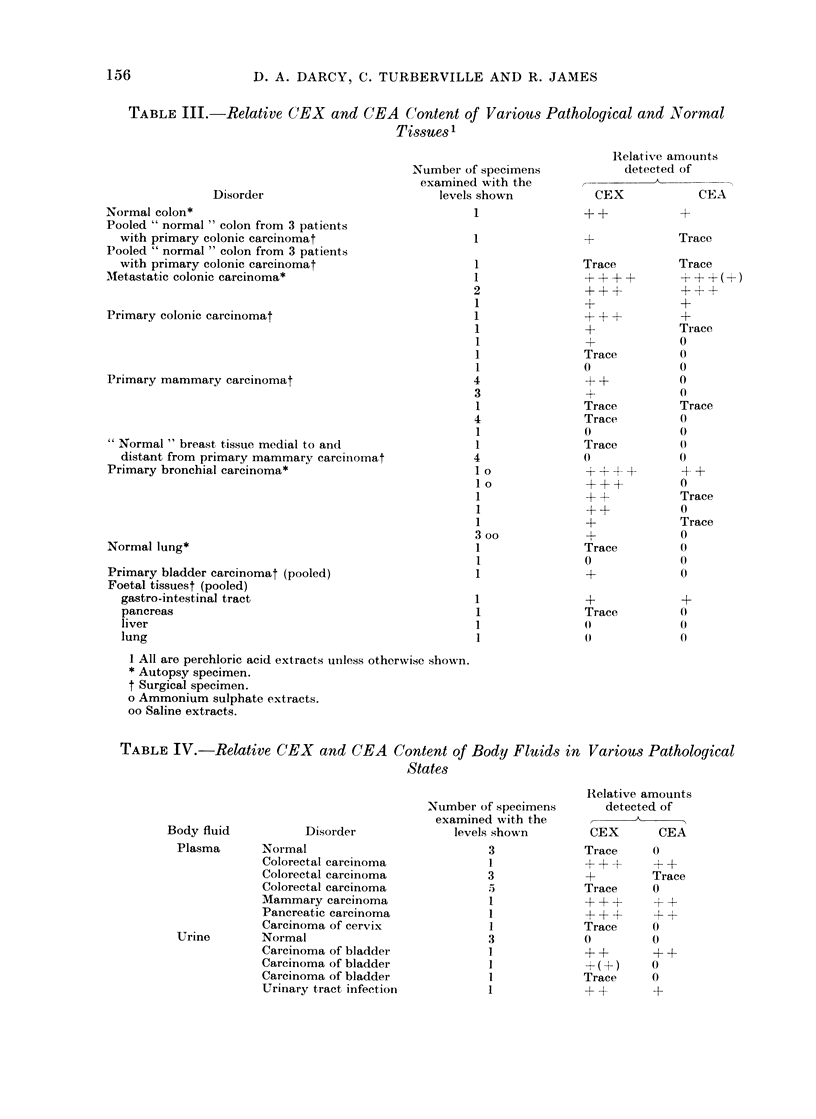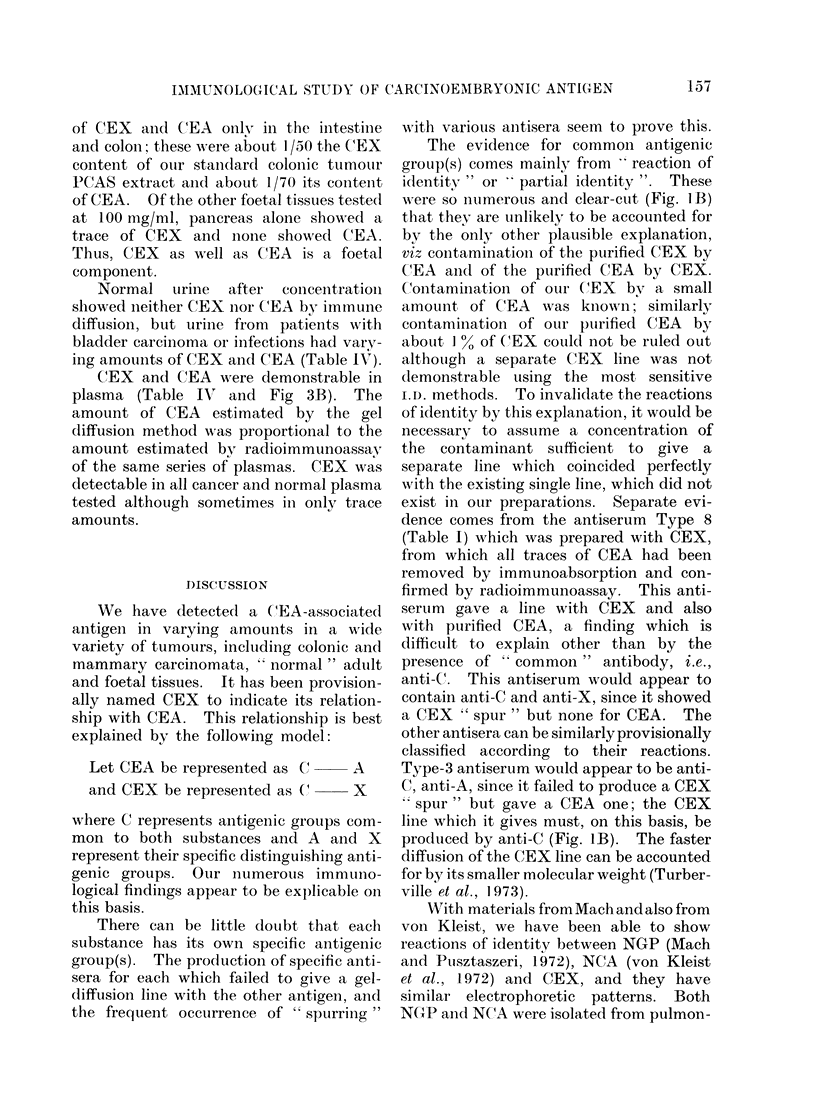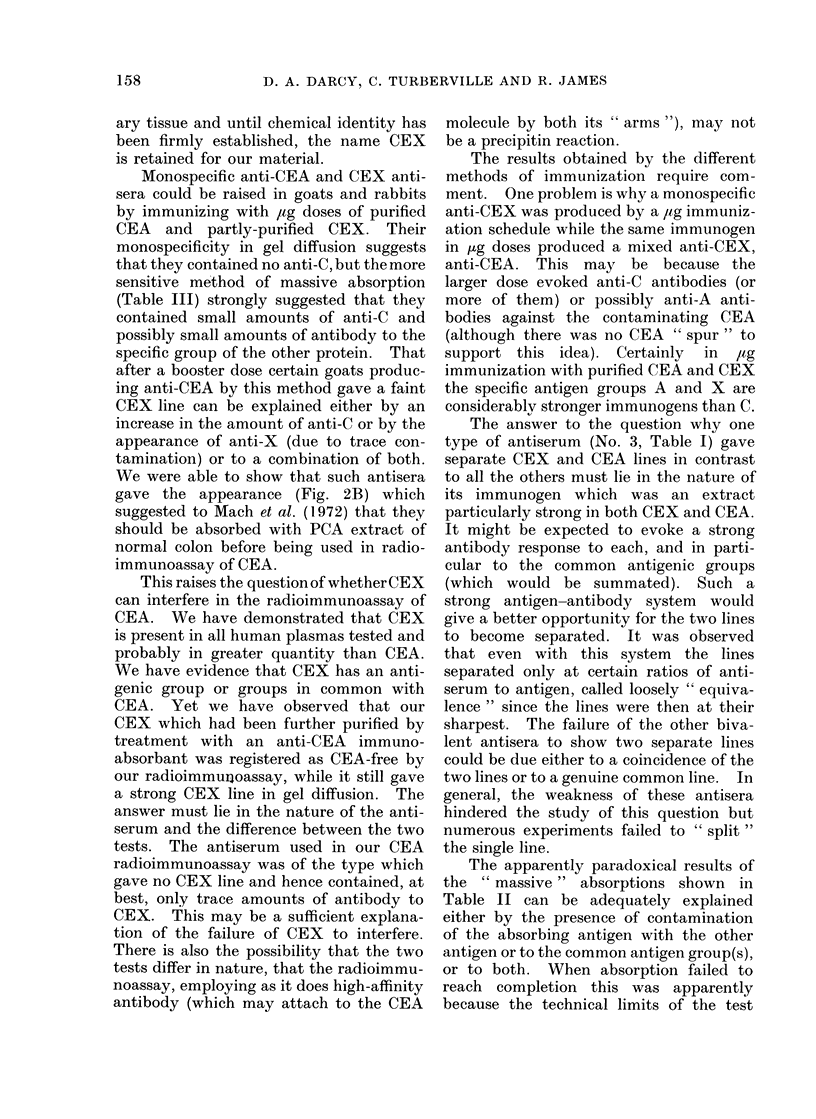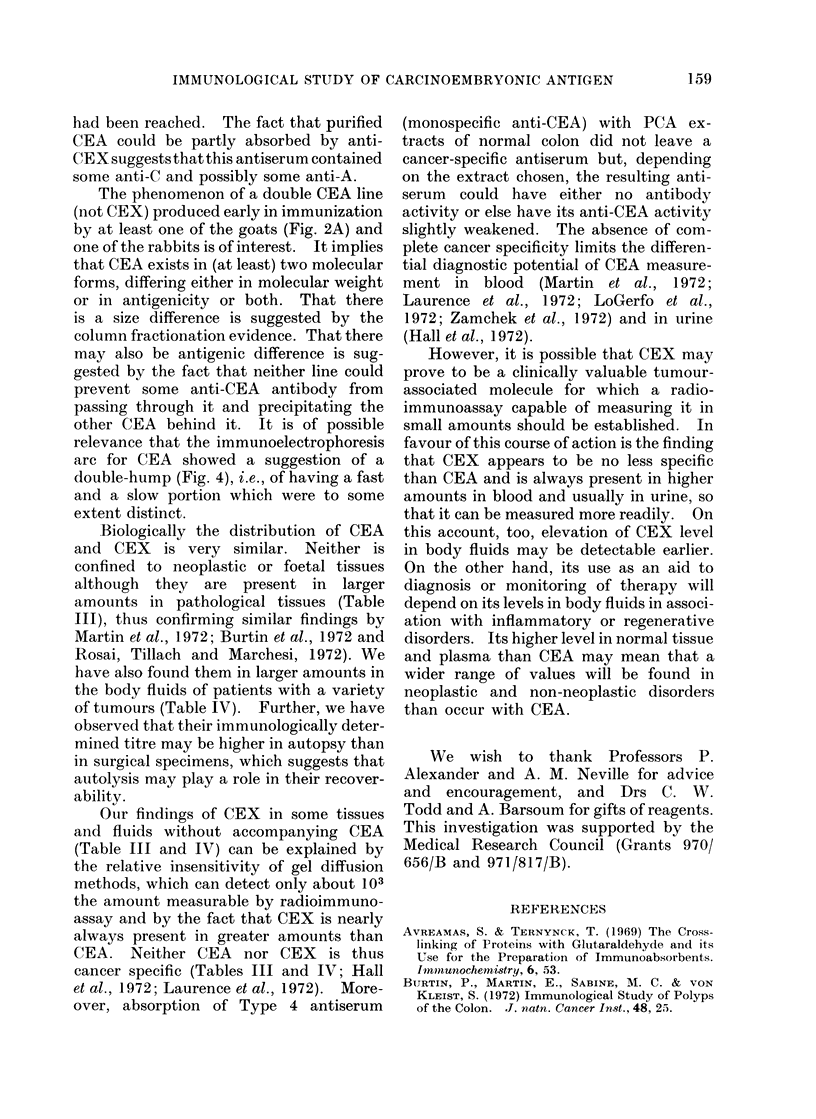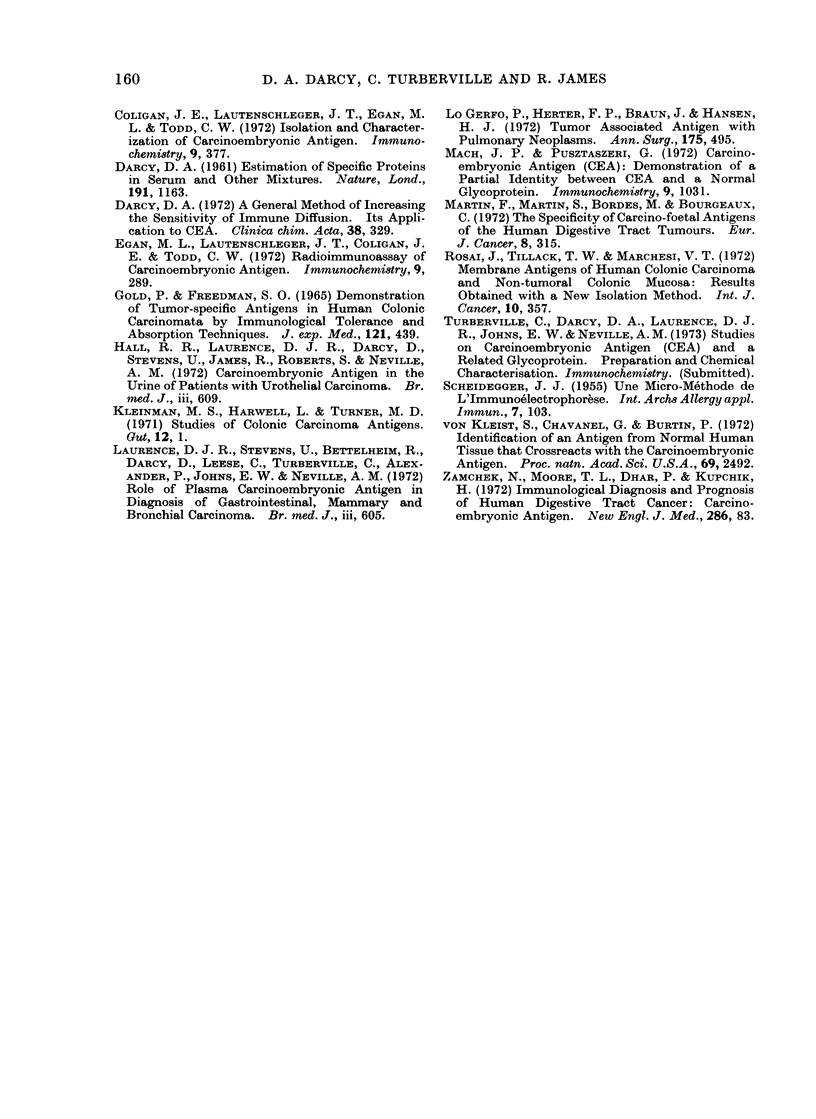# Immunological Study of Carcinoembryonic Antigen (CEA) and a Related Glycoprotein

**DOI:** 10.1038/bjc.1973.132

**Published:** 1973-08

**Authors:** D. A. Darcy, C. Turberville, R. James

## Abstract

**Images:**


*Br. J. Cancer* (1973) **28**, 147

### IMMUNOLOGICAL STUDY OF CARCINOEMBRYONIC ANTIGEN (CEA) AND A RELATED GLYCOPROTEIN

D. A. DARCY, C. TURBERVILLE AND R. JAMES

*From the Chester Beatty Research Institute at Clifton Avenue, Belmont, Sutton, Surrey, and Fulham Road, London, S.W.3*

Received 7 March 1973. Accepted 2 April 1973

**Summary.—A comparison has been made of the immunological properties of CEA (carcinoembryonic antigen) and another perchloric acid-soluble macromolecule which occurs in colonic and certain other carcinomata and which is here termed CEX. By using a variety of antisera it was shown that the two substances share common antigenic groups as well as having characteristic ones of their own. These latter groups have enabled the preparation of (a) antisera which give a gel diffusion line only with CEA and (b) an antiserum which gives a line only with CEX. No immunological difference could be found between CEX and the NGP of Mach or the NCA of von Kleist and Burtin. CEX was found in foetal gut, in plasma and associated with CEA in virtually all the tissues and fluids in which the latter occurs; the two appear to go hand-in-hand and no proof was found that CEX is either less or more cancer specific than CEA—it is merely found in greater quantity; neither substance showed absolute cancer specificity. The usefulness of a radioimmunoassay for CEX is discussed, and also the possibility of interference by CEX in the radioimmunoassay for CEA. Evidence of two molecular species of CEA has been found.**

SINCE Gold and Freedman (1965) first reported the existence of a glycoprotein antigen (CEA) in extracts of carcinomata of the colon and in the normal colon of human foetus of the first two trimesters of pregnancy, several authors have reported the existence of an associated protein antigen in these and other tissues which has been regarded, in general, as a more normal constituent of colon and certain other adult tissues. This protein has been given various names (NGP or normal glycoprotein, by Mach and Pusztaszeri, 1972; NCA or nonspecific cross-reacting antigen, by von Kleist, Chavanel and Burtin, 1972; Kleinman, Harwell and Turner (1971) report a similar protein), and it is not certain that they all refer to the same substance, although this is likely because of its striking relationship with CEA. Indeed, its existence was suggested in Gold's original publication.

Our purpose was to study these two substances and their relationship further, using immunological methods. Particular attention was paid to the "associated" protein provisionally called CEX, since there was a possibility that a comparison of its concentration with that of CEA in a given extract or blood might enhance the value of the information given by CEA assay.

### MATERIALS AND METHODS

*Antisera.*—Perhaps the most important part of this work was the preparation and comparison of various anti-CEA and anti-CEX (the CEA-associated protein) antisera. They were prepared by means of 3 injections of the antigen in complete Freund's adjuvant subcutaneously in the back, usually intrascapularly. The interval between the first and second injections was 2–4 weeks, and between the second and third 4–5 weeks.The first bleed was made about 2 weeks after the third injection. All bleeds were from the marginal ear vein of rabbits and from the jugular vein of goats. Occasionally a booster dose was given. The following antisera were made: (1) anti-saline extract of a human primary carcinoma of the colon made in 2 rabbits. The extract had 8·8 mg of protein per ml and each rabbit received 2·5 ml per injection; (2) anti-perchloric acid (PCA) extract of the same primary carcinoma of the colon in 2 rabbits. Each rabbit received 3·2 mg of the lyophilized extract per injection; (3) anti-PCA extract of a secondary colon carcinoma metastasis in the liver. The dose was 5 mg of the lyophilized extract per injection in 2 rabbits and 2·5 mg in another 2 rabbits; (4) anti-purified CEA prepared in 5 goats (one Gotenburg, 4 Irish mountain goats), each receiving 10 $\mu$g per injection according to Todd's method (Egan *et al.*, 1972); (5) anti-purified CEA prepared in 2 rabbits, each receiving 1 $\mu$g per injection; (6) anti-CEA prepared in 2 rabbits by injecting CEA-antibody precipitate lines cut from an Ouchterlony plate containing 1·5% I.D. agar gel. The precipitates were well washed in saline; (7) an anti-CEX prepared from the gel precipitates as for the preceding antiserum in 2 rabbits; (8) anti-CEX prepared in 2 rabbits by injecting the eluate of the perchloric acid extract of the same colon cancer used for antiserum No. 3 above, after treatment with an immunoadsorbant prepared by cross-linking the proteins of a goat anti-CEA serum with gluteraldehyde (Avreamas and Ternynck, 1969). Each rabbit received the equivalent of about 0·5 mg of the original extract per injection. This eluate was free of CEA, as shown by the ring test. Later, 2 more rabbits were injected with a similarly treated eluate which had been found to be CEA-free by radioimmunoassay; (9) anti-CEX prepared in 2 rabbits by injecting on each occasion 1 mg of a semi-purified CEX (1/41G) which originated from a colon carcinoma extract and was fractionated on Sepharose and Sephadex columns (see Turberville *et al.*, 1973); (10) anti-CEX prepared in 2 Salem goats injected on each occasion with 10 $\mu$g of the semi-purified CEX (1/41G).

*Absorption of antisera.*—Most anti-sera contained antibodies to human plasma proteins and these were removed by stepwise absorption with a pooled plasma from 10 donors, representing all major blood groups obtained from the National Blood Transfusion Centre. Next, antibodies to normal tissue antigens were removed by absorption with saline extract of normal human colon following Gold's procedure. These extracts showed little or no CEA or CEX by immune diffusion. Antisera types 4, 5, 6 and 7 required no absorption.

*Antigens.*—The principal antigens used *in vitro* were the perchloric acid extract of colon cancer metastatis in the liver used in (3) above (to be referred to as PCAS), and the purified CEA preparation made by the method of Todd (Coligan *et al.*, 1972). This involved gel filtration of the PCA extract on Sepharose 4B and then on Sephadex G200, the active fractions being detected with anti-CEA (4); CEX was prepared similarly but came off the columns in a different peak and was detected by antiserum No. 3 (Turberville *et al.*, 1973). Plasma was concentrated for testing by precipitating 5 ml with 1 mol/l perchloric acid, lyophilizing the water-dialysed supernatant and dissolving the resulting few mg of powder in 25 or 50 $\mu$l of saline. In some instances, the ICD (immune complex development) technique (Darcy, 1972) was employed to confirm the identity of very weak lines; this simply involved washing the gel free of soluble protein and then diffusing anti-rabbit IgG (or anti-goat, if appropriate) from a central well.

*Immunological methods.*—Double diffusion was carried out in 5-cm plastic petri dishes containing 1 mm of 1·5% agar gel in phosphate-buffered saline (pH 7). Wells were normally 5 mm in diameter with a distance between antigen and antibody wells (edge to edge) of 1·5, 3 or 5 mm. The method of Scheidegger (1955) was used for immunoelectrophoresis.

*Quantitation of tissue extracts and body fluids.*—The concentration of CEA and CEX in these was estimated semi-quantitatively by a modification of the quantitative Ouchterlony plate (Darcy, 1961). Our extract PCAS was taken as standard and arbitrarily assigned " +++ " for both CEA and CEX (of which it appeared to have approximately equal concentrations). All other extracts were compared with this extract using 5-mm diameter wells (holding about 20 $\mu$l) at a distance of 3 mm from the antiserum well. Extent of line migration (the measure of its antigen concentration) was compared by eye with that of the standard.

TABLE I.—*Antisera Raised Against CEA and CEX by Various Methods. Results were Those Obtained After Appropriate Absorptions**

| Number | Host | Immunogen | Dose level | Antibodies to CEA | Antibodies to CEX | One or 2 I.D. lines with PCAS† |
|---|---|---|---|---|---|---|
| 1 | Rabbit | Saline extract of primary colonic carcinoma | mg | + | + | One |
| 2 | Rabbit | PCA extract of primary colonic carcinoma | mg | + | + | One |
| 3 | Rabbit | PCA extract of metastatic colonic carcinoma | mg | + | + | Two |
| 4 | Goat | Purified CEA | μg | + | 0 | One |
| 5 | Rabbit | Purified CEA | μg | + | 0 | One |
| 6 | Rabbit | CEA gel-precipitate lines | mg | + | + | One |
| 7 | Rabbit | CEX gel-precipitate lines | mg | + | + | Equivocal |
| 8 | Rabbit | CEX purified by immuno-absorption | mg | + | + | One |
| 9 | Rabbit | CEX purified by column fractionation | mg | + | + | One |
| 10 | Goat | CEX purified by column fractionation | μg | 0 | + | One |

* See Methods.

† PCA extract of colonic carcinoma metastases.

### RESULTS

*Properties of the variously-prepared antisera (Table 1)*

(1) *Rabbit antisera to saline extracts of primary colon carcinoma.*—These antisera were unsatisfactorily weak, requiring concentration before use, probably because the original tumour extract was weak in CEX and particularly in CEA. After absorption they yielded a single line in gel diffusion with tumour extracts. When purified CEX and CEA were run simultaneously against them each antigen gave a single line which met the other in a reaction of identity.

(2) *Rabbit antisera to PCA extracts of primary colon carcinoma.*—These proved identical to the above (Type 1) but were somewhat stronger.

(3) *Rabbit antisera to PCA extracts of secondary colon carcinoma.*—These were the most interesting and useful antisera and have proved of value in estimating the relative amounts of CEA and CEX in column fractions using a modification of Darcy (1961). After absorption they yielded two strong Ouchterlony lines with the extract which gave rise to them (PCAS), the slower-diffusing of which was shown to be CEA while the faster-moving line was ascribed to the unknown antigen which was called CEX (Fig. 1A).

The second important property of these antisera was that their CEA and CEX lines *both* gave a reaction of identity with the single line obtained with purified CEA (Fig. 1B). The absence of " spurring " of the CEX line suggested that the CEA molecules not only shared antigens with CEX but that, so far as these antisera could indicate, CEX had no peculiar antigenic groupings; this was later disproved by other antisera (see below). When CEX was substituted for CEA in the configuration shown in Fig. 1B it produced a single line which gave an identity reaction only with the faster-diffusing line.

By means of these (Type 3) antisera it was shown that samples of CEA from various sources (the laboratories of Gold, Todd, our own, and other workers) gave reactions of identity but that traces of contaminants were revealed in most of them when the antisera were used in the unabsorbed state.

It is of interest that the two rabbits which received the smaller antigen dose gave less useful antisera in that their CEX and CEA lines were hardly separable (indicating a higher ratio of anti-CEX to anti-CEA) and they had more extraneous antibody to be absorbed.

(4) *Goat anti-purified CEA.*—A single line was obtained in gel diffusion with

Fig. 1A

Fig. 1B

FIG. 1.—Ouchterlony plates showing reactions of various CEA and CEX preparations with various antisera.

A. Perchloric acid extract of a secondary colon carcinoma (PCAS) gives 2 lines with an antiserum (Type 3) to itself, whether unabsorbed (top left) or absorbed (top right). It gives a single line with anti-CEA (Type 4) which shows a reaction of identity with the slower diffusing line which was therefore called the CEA line while the other was called the CEX line. Absorption of anti-PCAS made little difference.

B. Purified CEA gives a reaction of identity with both the CEA and CEX lines.

purified CEA and with the crude PCA tumour extract (PCAS). No line was obtainable with CEX. However, later in immunization, after a booster dose of 1 $\mu$g of CEA, 2 of the 5 goats developed a CEX line; this was faint in one but moderately strong on the other (the Gotenburg goat). The identity of this CEX line (which diffused ahead of the CEA line) was confirmed not only by a reaction of identity but also by the use of CEX and monospecific anti-CEX serum (Type 10).

An early antiserum (3G1) from one of the Irish goats gave a double CEA line (with both purified CEA and with PCAS) and which by its cross-reactions with others of these goat anti-CEA sera strongly suggested that they also would demonstrate this phenomenon given the correct antigen–antibody ratio (Fig. 2A). None of these antisera gave a CEX line. A later (purer) CEA preparation, made by " cutting " the column peaks of CEA more narrowly, showed the same 2 CEA lines but much closer together and with their positions reversed. This indicated that the faster diffusing component had been reduced in concentration.

(5) *Rabbit anti-purified CEA.*—When bled 2 weeks after the last injection one of the resulting sera gave no line with CEX or CEA, but the second gave a line with CEA and not with CEX, thus appearing equivalent to the goat anti-CEA (Type 4).

(6) *Rabbit anti-CEA gel precipitate lines.*—These antisera were weak. The stronger showed essentially a reaction of identity between CEX and CEA (which here gave a double line) but there was a faint CEA " spur " indicating some specific anti-CEA antibodies. The 2 CEA lines given by this antiserum were compared with those given by antiserum 3G1 (Type 4 above), but it was impossible to be certain that they were the same, even though there was an overall reaction of identity.

(7) *Rabbit anti-CEX gel precipitate lines.*—Both antisera were weak but the stronger one, when concentrated about 10 times, was found to have antibodies to CEA as well as to CEX. The presence of specific anti-CEX antibodies was indicated by a " spur ".

(8) *Rabbit anti-CEX (CEX purified by immunoabsorption).*—These antisera all had antibodies to CEA as well as to CEX. One of the 2 antisera prepared against the CEX which had been found CEA-free by radioimmunoassay showed the CEX line giving a spur over the CEA line but not *vice versa*, thus indicating the presence of specific anti-CEX antibody.

(9) *Rabbit anti-CEX (CEX purified by column fractionation).*—Antisera from both rabbits had antibodies to CEA as well as to CEX and gave a clear-cut " reaction of partial identity " between the 2 antigens, the CEX line showing a spur past the CEA line, again indicating the presence of specific anti-CEX antibodies but no specific anti-CEA ones.

(10) *Goat anti-CEX (CEX purified by column fractionation).*—The only difference in procedure from No. 9 was that the goats received $\mu$g quantities of the antigen compared with mg amounts injected into the rabbits. Antisera from both goats gave several lines. In one goat these lines were found by absorption to be produced entirely by impurities in the CEX preparation. However, absorption of the antiserum from the other goat with plasma and normal colon left antibodies which reacted with CEX (to give a single line) but not with CEA at any concentration. The goal of producing a monospecific anti-CEX antiserum had apparently been achieved.

### *Absorption studies*

These are summarized in Table II. Type 3 antiserum (anti-PCAS) was further absorbed with purified CEA, purified CEX and the crude PCAS. As would be expected, no lines were obtained with the antiserum after absorption with PCAS or CEA (which reacted with the anti-CEX antibodies (Fig. 1B). Absorption with purified CEX removed the CEX-line and

Fig. 2A

Fig. 2B

FIG. 2.—A. Purified CEA gives 2 lines with goat anti-CEA (1) and suggestively with anti-CEA from goats (2) and (3). All are Type 4 antisera.

B. CEX gives a reaction of partial identity with CEA if an unabsorbed " impure " Type 4 anti-CEA is employed (left) but not when the antiserum is absorbed with normal colon PCA extract (right).

TABLE II.—*Results of Absorption of Antisera and Antigens*

| Substance absorbed | Absorbant | Substances removed by: Moderate absorption | Substances removed by: Massive absorption |
|---|---|---|---|
| Antiserum Type 3 (anti-CEX, anti-CEA) | CEA | a'CEX and a'CEA | |
| | CEX | a'CEX only | a'CEX and a'CEA |
| | PCAS* | a'CEX and a'CEA | |
| Antiserum Type 4 (anti-CEA) | PCAS | a'CEA | |
| | PCA normal colon 1 | a'CEA | |
| | PCA normal colon 2 | a'CEA | |
| | PCA normal colon 3 | Little or no effect | |
| | CEX | | a'CEA |
| Antiserum Type 10 (anti-CEX) | CEA | | Most a'CEX |
| CEA | a'CEA (4) | CEA, including its CEX-reactivity | |
| | a'CEX (10) | Little or no effect | Some CEA |
| CEX | a'CEA (4) | CEA contaminant and some CEX | All CEX |
| PCAS* | a'CEA (4) | CEA | All CEX and CEA |
| | a'CEA (10) | Some CEX | All CEX, some CEA |

* PCA extract of colonic carcinoma metastases.

left the CEA line slightly weakened. Massive absorption with CEX removed the CEA line also; this was not a dilution effect. The reasons for it are discussed below.

Type 4 antiserum (anti-CEA, no anti-CEX) was absorbed completely by PCAS and also by massive amounts of CEX preparations (all of which contained traces of CEA). We attempted to see if absorption by PCA extracts of normal colon would remove an anti-normal component and leave a specific anti-cancer CEA. Three different " normal " colon PCA extracts were employed which contained differing amounts of CEA and CEX (Table III). The strongest was an autopsy specimen which contained CEA and CEX at 1/20 and 1/10 respectively of the level in our tumour extract PCAS. The next strongest was from the uninvolved colon of patients with colonic carcinomata. Absorption with either of these extracts removed all antibody activity, whereas absorption with the weak third extract (also from colon carcinoma patients) merely weakened the CEA line. It is apparent therefore that the outcome of the absorption can be determined by the selection of the absorbing " normal " extracts. It is also of some interest that we were unable to find a PCA extract of " normal " colon which was completely free of CEA and CEX. We could readily produce the picture obtained by Mach and Pusztaszeri (1972) by using an impure anti-CEA, *i.e.*, one of our goat anti-CEA which contained some anti-CEX, before and after absorption with the " weak " colon extract (Fig. 2B). This confirms the correctness of the view of these authors that it is necessary to absorb this type of antiserum with an extract of normal colon.

Type-10 antiserum (anti-CEX) could be absorbed only by massive amounts of CEA. A faint CEX line was still obtained at the technical limits.

Absorption of purified CEA by anti-CEA had as its purpose to see if any of its CEX reactivity (Fig. 1B) would remain; it did not. Nor was it possible to demonstrate any CEX in purified CEA by the most sensitive gel methods. Absorption

Fig. 3A

Fig. 3B

FIG. 3.—A. Reaction of identity between CEX and NGP using anti-CEX (Type 10), and also between the anti-CEX and anti-NCA although this is partly obscured by impurities.
B. PCA extracts of cancer plasma show both CEA and CEX lines (P3 and P1) or only the CEX line (P2). Before extraction, P1 and P3 contained 1000 ng per ml of CEA while P2 contained 136 ng per ml. Lines were enhanced by ICD (Darcy, 1972).

of CEA with massive amounts of anti-CEX weakened the CEA line.

Absorption of CEX preparations with anti-CEA in massive amounts removed all CEX. This difference from the preceding experiment almost certainly reflects merely the relative strengths of the preparations. Absorption of the crude colonic carcinoma extract (PCAS) with anti-CEA removed the CEA line which it gave with antiserum Type 3 but left its CEX line. Massive absorption removed both lines. When it was absorbed with anti-CEX the reverse tended to happen but fell short of completion because of the weakness of the antiserum.

FIG. 4.—Immunoelectrophoresis of CEA (top well) and CEX (bottom well) with antiserum against both (Type 1) in the centre trough.

*Comparison of CEX with the CEA-associated protein of other authors*

Fig. 4 shows an immunoelectrophoresis of CEA along with CEX using rabbit antiserum Type 1 (Table I). The CEX gives a short arc which is entirely in the $\beta$-region (it encircles the origin, mainly on its $\gamma$-side). The CEA arc is a long one which extends from the $\beta$-region to the $\alpha$-region and there is a suggestion that it is a double-humped arc. These results were obtained on 1% I.D. Oxoid agar at pH 8·2. The same picture was, in its general features, obtained by others (Mach and Pusztaszeri, 1972; von Kleist *et al.*, 1972).

An antiserum to her nonspecific cross-reacting antigen (NCA) was kindly sent by Dr Sabine von Kleist. This gave a reaction of identity with our goat anti-CEX serum, using as antigen our purified CEX. Likewise, Dr Mach kindly sent us a sample of his purified NGP (normal glycoprotein). This gave a reaction of identity with our purified CEX using Type 10 antiserum (Fig. 3A).

*Occurrence of CEA and CEX in other tissues and fluids*

Our findings are shown in Table III and IV. The quantitative data are only approximate and the numbers of " + " are more logarithmic than arithmetic. Primary colonic carcinomata have, as a group, less CEX and CEA than their metastases; they also have a higher ratio of CEX to CEA. Normal colon obtained at autopsy can have a CEX content about 1/10 that of our standard metastatic extract (PCAS) and a CEA content about 1/20 of it. The other colon specimens, obtained at operation, contained less of these antigens.

Primary mammary carcinomata contain more CEX than CEA (which by this method could not be detected). The amounts of CEX are comparable with that in primary colonic carcinomata. The occurrence of CEX in normal breast tissue was uncertain.

Primary bronchial carcinomata also contain a preponderance of CEX over CEA. Normal lung tissue extracts showed little or no CEX or CEA at the tested concentrations. The extract of pooled primary bladder carcinoma showed (at 50 mg/ml) some CEX but no CEA.

Foetal tissues from a pool, ranging in gestational age from 10 to 23 weeks and tested at the high concentration of 100 mg/ml of the lyophilized dialysed PCA extract, showed measurable amounts

TABLE III.—*Relative CEX and CEA Content of Various Pathological and Normal Tissues*[1]

| Disorder | Number of specimens examined with the levels shown | Relative amounts detected of CEX | CEA |
|---|---|---|---|
| Normal colon* | 1 | ++ | + |
| Pooled " normal " colon from 3 patients with primary colonic carcinoma† | 1 | + | Trace |
| Pooled " normal " colon from 3 patients with primary colonic carcinoma† | 1 | Trace | Trace |
| Metastatic colonic carcinoma* | 1 | ++++ | +++(+) |
| | 2 | +++ | +++ |
| | 1 | + | + |
| Primary colonic carcinoma† | 1 | +++ | + |
| | 1 | + | Trace |
| | 1 | + | 0 |
| | 1 | Trace | 0 |
| | 1 | 0 | 0 |
| Primary mammary carcinoma† | 4 | ++ | 0 |
| | 3 | + | 0 |
| | 1 | Trace | Trace |
| | 4 | Trace | 0 |
| | 1 | 0 | 0 |
| " Normal " breast tissue medial to and distant from primary mammary carcinoma† | 1 | Trace | 0 |
| | 4 | 0 | 0 |
| Primary bronchial carcinoma* | 1 o | ++++ | ++ |
| | 1 o | +++ | 0 |
| | 1 | ++ | Trace |
| | 1 | ++ | 0 |
| | 1 | + | Trace |
| | 3 oo | + | 0 |
| Normal lung* | 1 | Trace | 0 |
| | 1 | 0 | 0 |
| Primary bladder carcinoma† (pooled) | 1 | + | 0 |
| Foetal tissues† (pooled) | | | |
| gastro-intestinal tract | 1 | + | + |
| pancreas | 1 | Trace | 0 |
| liver | 1 | 0 | 0 |
| lung | 1 | 0 | 0 |

1 All are perchloric acid extracts unless otherwise shown.
* Autopsy specimen.
† Surgical specimen.
o Ammonium sulphate extracts.
oo Saline extracts.

TABLE IV.—*Relative CEX and CEA Content of Body Fluids in Various Pathological States*

| Body fluid | Disorder | Number of specimens examined with the levels shown | Relative amounts detected of CEX | CEA |
|---|---|---|---|---|
| Plasma | Normal | 3 | Trace | 0 |
| | Colorectal carcinoma | 1 | +++ | ++ |
| | Colorectal carcinoma | 3 | + | Trace |
| | Colorectal carcinoma | 5 | Trace | 0 |
| | Mammary carcinoma | 1 | +++ | ++ |
| | Pancreatic carcinoma | 1 | +++ | ++ |
| | Carcinoma of cervix | 1 | Trace | 0 |
| Urine | Normal | 3 | 0 | 0 |
| | Carcinoma of bladder | 1 | ++ | ++ |
| | Carcinoma of bladder | 1 | +(+) | 0 |
| | Carcinoma of bladder | 1 | Trace | 0 |
| | Urinary tract infection | 1 | ++ | + |

of CEX and CEA only in the intestine and colon; these were about 1/50 the CEX content of our standard colonic tumour PCAS extract and about 1/70 its content of CEA. Of the other foetal tissues tested at 100 mg/ml, pancreas alone showed a trace of CEX and none showed CEA. Thus, CEX as well as CEA is a foetal component.

Normal urine after concentration showed neither CEX nor CEA by immune diffusion, but urine from patients with bladder carcinoma or infections had varying amounts of CEX and CEA (Table IV).

CEX and CEA were demonstrable in plasma (Table IV and Fig 3B). The amount of CEA estimated by the gel diffusion method was proportional to the amount estimated by radioimmunoassay of the same series of plasmas. CEX was detectable in all cancer and normal plasma tested although sometimes in only trace amounts.

### DISCUSSION

We have detected a CEA-associated antigen in varying amounts in a wide variety of tumours, including colonic and mammary carcinomata, " normal " adult and foetal tissues. It has been provisionally named CEX to indicate its relationship with CEA. This relationship is best explained by the following model:

Let CEA be represented as C —— A

and CEX be represented as C —— X

where C represents antigenic groups common to both substances and A and X represent their specific distinguishing antigenic groups. Our numerous immunological findings appear to be explicable on this basis.

There can be little doubt that each substance has its own specific antigenic group(s). The production of specific antisera for each which failed to give a gel-diffusion line with the other antigen, and the frequent occurrence of " spurring " with various antisera seem to prove this.

The evidence for common antigenic group(s) comes mainly from " reaction of identity " or " partial identity ". These were so numerous and clear-cut (Fig. 1B) that they are unlikely to be accounted for by the only other plausible explanation, *viz* contamination of the purified CEX by CEA and of the purified CEA by CEX. Contamination of our CEX by a small amount of CEA was known; similarly contamination of our purified CEA by about 1% of CEX could not be ruled out although a separate CEX line was not demonstrable using the most sensitive I.D. methods. To invalidate the reactions of identity by this explanation, it would be necessary to assume a concentration of the contaminant sufficient to give a separate line which coincided perfectly with the existing single line, which did not exist in our preparations. Separate evidence comes from the antiserum Type 8 (Table I) which was prepared with CEX, from which all traces of CEA had been removed by immunoabsorption and confirmed by radioimmunoassay. This antiserum gave a line with CEX and also with purified CEA, a finding which is difficult to explain other than by the presence of " common " antibody, *i.e.*, anti-C. This antiserum would appear to contain anti-C and anti-X, since it showed a CEX " spur " but none for CEA. The other antisera can be similarly provisionally classified according to their reactions. Type-3 antiserum would appear to be anti-C, anti-A, since it failed to produce a CEX " spur " but gave a CEA one; the CEX line which it gives must, on this basis, be produced by anti-C (Fig. 1B). The faster diffusion of the CEX line can be accounted for by its smaller molecular weight (Turberville *et al.*, 1973).

With materials from Mach and also from von Kleist, we have been able to show reactions of identity between NGP (Mach and Pusztaszeri, 1972), NCA (von Kleist *et al.*, 1972) and CEX, and they have similar electrophoretic patterns. Both NGP and NCA were isolated from pulmonary tissue and until chemical identity has been firmly established, the name CEX is retained for our material.

Monospecific anti-CEA and CEX antisera could be raised in goats and rabbits by immunizing with μg doses of purified CEA and partly-purified CEX. Their monospecificity in gel diffusion suggests that they contained no anti-C, but the more sensitive method of massive absorption (Table III) strongly suggested that they contained small amounts of anti-C and possibly small amounts of antibody to the specific group of the other protein. That after a booster dose certain goats producing anti-CEA by this method gave a faint CEX line can be explained either by an increase in the amount of anti-C or by the appearance of anti-X (due to trace contamination) or to a combination of both. We were able to show that such antisera gave the appearance (Fig. 2B) which suggested to Mach *et al.* (1972) that they should be absorbed with PCA extract of normal colon before being used in radioimmunoassay of CEA.

This raises the question of whether CEX can interfere in the radioimmunoassay of CEA. We have demonstrated that CEX is present in all human plasmas tested and probably in greater quantity than CEA. We have evidence that CEX has an antigenic group or groups in common with CEA. Yet we have observed that our CEX which had been further purified by treatment with an anti-CEA immunoabsorbant was registered as CEA-free by our radioimmunoassay, while it still gave a strong CEX line in gel diffusion. The answer must lie in the nature of the antiserum and the difference between the two tests. The antiserum used in our CEA radioimmunoassay was of the type which gave no CEX line and hence contained, at best, only trace amounts of antibody to CEX. This may be a sufficient explanation of the failure of CEX to interfere. There is also the possibility that the two tests differ in nature, that the radioimmunoassay, employing as it does high-affinity antibody (which may attach to the CEA molecule by both its " arms "), may not be a precipitin reaction.

The results obtained by the different methods of immunization require comment. One problem is why a monospecific anti-CEX was produced by a μg immunization schedule while the same immunogen in μg doses produced a mixed anti-CEX, anti-CEA. This may be because the larger dose evoked anti-C antibodies (or more of them) or possibly anti-A antibodies against the contaminating CEA (although there was no CEA " spur " to support this idea). Certainly in μg immunization with purified CEA and CEX the specific antigen groups A and X are considerably stronger immunogens than C.

The answer to the question why one type of antiserum (No. 3, Table I) gave separate CEX and CEA lines in contrast to all the others must lie in the nature of its immunogen which was an extract particularly strong in both CEX and CEA. It might be expected to evoke a strong antibody response to each, and in particular to the common antigenic groups (which would be summated). Such a strong antigen–antibody system would give a better opportunity for the two lines to become separated. It was observed that even with this system the lines separated only at certain ratios of antiserum to antigen, called loosely " equivalence " since the lines were then at their sharpest. The failure of the other bivalent antisera to show two separate lines could be due either to a coincidence of the two lines or to a genuine common line. In general, the weakness of these antisera hindered the study of this question but numerous experiments failed to " split " the single line.

The apparently paradoxical results of the " massive " absorptions shown in Table II can be adequately explained either by the presence of contamination of the absorbing antigen with the other antigen or to the common antigen group(s), or to both. When absorption failed to reach completion this was apparently because the technical limits of the test had been reached. The fact that purified CEA could be partly absorbed by anti-CEX suggests that this antiserum contained some anti-C and possibly some anti-A.

The phenomenon of a double CEA line (not CEX) produced early in immunization by at least one of the goats (Fig. 2A) and one of the rabbits is of interest. It implies that CEA exists in (at least) two molecular forms, differing either in molecular weight or in antigenicity or both. That there is a size difference is suggested by the column fractionation evidence. That there may also be antigenic difference is suggested by the fact that neither line could prevent some anti-CEA antibody from passing through it and precipitating the other CEA behind it. It is of possible relevance that the immunoelectrophoresis arc for CEA showed a suggestion of a double-hump (Fig. 4), *i.e.*, of having a fast and a slow portion which were to some extent distinct.

Biologically the distribution of CEA and CEX is very similar. Neither is confined to neoplastic or foetal tissues although they are present in larger amounts in pathological tissues (Table III), thus confirming similar findings by Martin *et al.*, 1972; Burtin *et al.*, 1972 and Rosai, Tillach and Marchesi, 1972). We have also found them in larger amounts in the body fluids of patients with a variety of tumours (Table IV). Further, we have observed that their immunologically determined titre may be higher in autopsy than in surgical specimens, which suggests that autolysis may play a role in their recoverability.

Our findings of CEX in some tissues and fluids without accompanying CEA (Table III and IV) can be explained by the relative insensitivity of gel diffusion methods, which can detect only about $10^3$ the amount measurable by radioimmunoassay and by the fact that CEX is nearly always present in greater amounts than CEA. Neither CEA nor CEX is thus cancer specific (Tables III and IV; Hall *et al.*, 1972; Laurence *et al.*, 1972). Moreover, absorption of Type 4 antiserum (monospecific anti-CEA) with PCA extracts of normal colon did not leave a cancer-specific antiserum but, depending on the extract chosen, the resulting antiserum could have either no antibody activity or else have its anti-CEA activity slightly weakened. The absence of complete cancer specificity limits the differential diagnostic potential of CEA measurement in blood (Martin *et al.*, 1972; Laurence *et al.*, 1972; LoGerfo *et al.*, 1972; Zamchek *et al.*, 1972) and in urine (Hall *et al.*, 1972).

However, it is possible that CEX may prove to be a clinically valuable tumour-associated molecule for which a radioimmunoassay capable of measuring it in small amounts should be established. In favour of this course of action is the finding that CEX appears to be no less specific than CEA and is always present in higher amounts in blood and usually in urine, so that it can be measured more readily. On this account, too, elevation of CEX level in body fluids may be detectable earlier. On the other hand, its use as an aid to diagnosis or monitoring of therapy will depend on its levels in body fluids in association with inflammatory or regenerative disorders. Its higher level in normal tissue and plasma than CEA may mean that a wider range of values will be found in neoplastic and non-neoplastic disorders than occur with CEA.

We wish to thank Professors P. Alexander and A. M. Neville for advice and encouragement, and Drs C. W. Todd and A. Barsoum for gifts of reagents. This investigation was supported by the Medical Research Council (Grants 970/656/B and 971/817/B).

### REFERENCES

AVREAMAS, S. & TERNYNCK, T. (1969) The Cross-linking of Proteins with Glutaraldehyde and its Use for the Preparation of Immunoabsorbents. *Immunochemistry*, **6**, 53.

BURTIN, P., MARTIN, E., SABINE, M. C. & VON KLEIST, S. (1972) Immunological Study of Polyps of the Colon. *J. natn. Cancer Inst.*, **48**, 25.

COLIGAN, J. E., LAUTENSCHLEGER, J. T., EGAN, M. L. & TODD, C. W. (1972) Isolation and Characterization of Carcinoembryonic Antigen. *Immunochemistry*, **9**, 377.

DARCY, D. A. (1961) Estimation of Specific Proteins in Serum and Other Mixtures. *Nature, Lond.*, **191**, 1163.

DARCY, D. A. (1972) A General Method of Increasing the Sensitivity of Immune Diffusion. Its Application to CEA. *Clinica chim. Acta*, **38**, 329.

EGAN, M. L., LAUTENSCHLEGER, J. T., COLIGAN, J. E. & TODD, C. W. (1972) Radioimmunoassay of Carcinoembryonic Antigen. *Immunochemistry*, **9**, 289.

GOLD, P. & FREEDMAN, S. O. (1965) Demonstration of Tumor-specific Antigens in Human Colonic Carcinomata by Immunological Tolerance and Absorption Techniques. *J. exp. Med.*, **121**, 439.

HALL, R. R., LAURENCE, D. J. R., DARCY, D., STEVENS, U., JAMES, R., ROBERTS, S. & NEVILLE, A. M. (1972) Carcinoembryonic Antigen in the Urine of Patients with Urothelial Carcinoma. *Br. med. J.*, iii, 609.

KLEINMAN, M. S., HARWELL, L. & TURNER, M. D. (1971) Studies of Colonic Carcinoma Antigens. *Gut*, **12**, 1.

LAURENCE, D. J. R., STEVENS, U., BETTELHEIM, R., DARCY, D., LEESE, C., TURBERVILLE, C., ALEXANDER, P., JOHNS, E. W. & NEVILLE, A. M. (1972) Role of Plasma Carcinoembryonic Antigen in Diagnosis of Gastrointestinal, Mammary and Bronchial Carcinoma. *Br. med. J.*, iii, 605.

LO GERFO, P., HERTER, F. P., BRAUN, J. & HANSEN, H. J. (1972) Tumor Associated Antigen with Pulmonary Neoplasms. *Ann. Surg.*, **175**, 495.

MACH, J. P. & PUSZTASZERI, G. (1972) Carcinoembryonic Antigen (CEA): Demonstration of a Partial Identity between CEA and a Normal Glycoprotein. *Immunochemistry*, **9**, 1031.

MARTIN, F., MARTIN, S., BORDES, M. & BOURGEAUX, C. (1972) The Specificity of Carcino-foetal Antigens of the Human Digestive Tract Tumours. *Eur. J. Cancer*, **8**, 315.

ROSAI, J., TILLACK, T. W. & MARCHESI, V. T. (1972) Membrane Antigens of Human Colonic Carcinoma and Non-tumoral Colonic Mucosa: Results Obtained with a New Isolation Method. *Int. J. Cancer*, **10**, 357.

TURBERVILLE, C., DARCY, D. A., LAURENCE, D. J. R., JOHNS, E. W. & NEVILLE, A. M. (1973) Studies on Carcinoembryonic Antigen (CEA) and a Related Glycoprotein. Preparation and Chemical Characterisation. *Immunochemistry*. (Submitted).

SCHEIDEGGER, J. J. (1955) Une Micro-Méthode de L'Immunoélectrophorèse. *Int. Archs Allergy appl. Immun.*, **7**, 103.

VON KLEIST, S., CHAVANEL, G. & BURTIN, P. (1972) Identification of an Antigen from Normal Human Tissue that Crossreacts with the Carcinoembryonic Antigen. *Proc. natn. Acad. Sci. U.S.A.*, **69**, 2492.

ZAMCHEK, N., MOORE, T. L., DHAR, P. & KUPCHIK, H. (1972) Immunological Diagnosis and Prognosis of Human Digestive Tract Cancer: Carcinoembryonic Antigen. *New Engl. J. Med.*, **286**, 83.